# Termite Taxonomy, Challenges and Prospects: West Africa, A Case Example

**DOI:** 10.3390/insects10010032

**Published:** 2019-01-16

**Authors:** Judith Korb, Boris D. Kasseney, Yvonne Tété Cakpo, Robin H. Casalla Daza, Jean Norbert K. B. Gbenyedji, Mayouré Edith Ilboudo, Guy Josens, N’golo Abdoulaye Koné, Karen Meusemann, Abdoulaye B. Ndiaye, Simon Idoko Okweche, Michael Poulsen, Yves Roisin, Fernand Sankara

**Affiliations:** 1 Zoology I: Evolutionary Biology & Ecology, University of Freiburg, Hauptstrasse 1, 79104-Freiburg, Germany; casallar@uninorte.edu.co (R.H.C.D.); Karen.meusemann@biologie.uni-freiburg.de (K.M.); 2 Laboratory of Applied Entomology, Department of Zoology and Animal Biology, Faculty of Sciences, S/C University of Lomé, 1 BP 1515 Lomé 1, Togo; borisdodji@gmail.com (B.D.K.); koamigbenyedji@gmail.com (J.N.K.B.G.); 3 Laboratory of Applied Ecology, University of Abomey-Calavi, 03 BP 3908 Cotonou, Benin; ycakpo@gmail.com; 4 Departamento de Química y Biología, Universidad del Norte, Kilómetro 5 Antigua vía Puerto Colombia, 081007-Puerto Colombia, Colombia; 5 Institute of Science (IDS), University Ouaga I Pr Joseph KI-ZERBO (UO1PJKZ), O1 BP 2127 Ouagadougou 01, Burkina Faso; imaye78@yahoo.fr; 6 Ecologie végétale et biogéochimie, Université Libre de Bruxelles, Avenue F.D. Roosevelt 50, 1050 Bruxelles, Belgium; gjosens@ulb.ac.be; 7 Université Nangui Abrogoua, UFR des Sciences de la Nature (UFR SN), Unité de Recherche en Ecologie et Biodiversité (UREB) & Station de Recherche en Ecologie du Parc National de la Comoé, 28 BP 847 Abidjan 28, Cote d’Ivoire; ngolo197804@yahoo.fr; 8 Laboratoire de Zoologie des Invertébrés Terrestres, IFAN B. P. 206 Dakar, Senegal; abdoulayeb.ndiaye@ucad.edu.sn; 9 Department of Forestry and Wildlife Resources Management, University of Calabar, Calabar Cross River State, P.M.B 1123 Calabar, Nigeria; idoko.sim@gmail.com; 10 Department of Biology, Section for Ecology and Evolution, University of Copenhagen, Universitetsparken 15, 2100 Copenhagen East, Denmark; MPoulsen@bio.ku.dk; 11 Evolutionary Biology & Ecology, Université Libre de Bruxelles, Avenue F.D. Roosevelt 50, B-150 Brussels, Belgium; yroisin@ulb.ac.be; 12 Institut du Développement Rural, Université Nazi Boni, 01BP 1091 Bobo 01, Burkina Faso; ferdisank2005@yahoo.fr

**Keywords:** Africa, barcoding, ecology, Isoptera, social insect, taxonomy, termite

## Abstract

Termites are important ecosystem engineers. Yet they are often difficult to identify due to the lack of reliable species-specific morphological traits for many species, which hampers ecological research. Recently, termitologists working with West African termites (West African Termite Taxonomy Initiative) convened for a workshop with the aim of beginning to address this problem. Repeated determination of the same termite samples by the most renowned taxonomists for West African termites identified the huge scale of the problem, as less than 10% of all species could be unambiguously determined to the species level. Intensive discussions and comparisons increased the identification success to around 25% at the end of the workshop. Yet many groups remained problematic and molecular markers and barcoding techniques combined with species delimitation approaches will be needed to help resolve these existing taxonomic problems. Based on the outcome of this workshop, we propose concerted initiatives to address termite taxonomy on a global scale. We are convinced that dedicated workshops on regional taxonomy that follow a similar structured approach, with repeated determination of the same sample, will help overcome the difficulties that termite taxonomy faces. This initiative can also serve as a blueprint for other taxonomical groups that are difficult to identify.

## 1. Introduction

**Figure 1:**
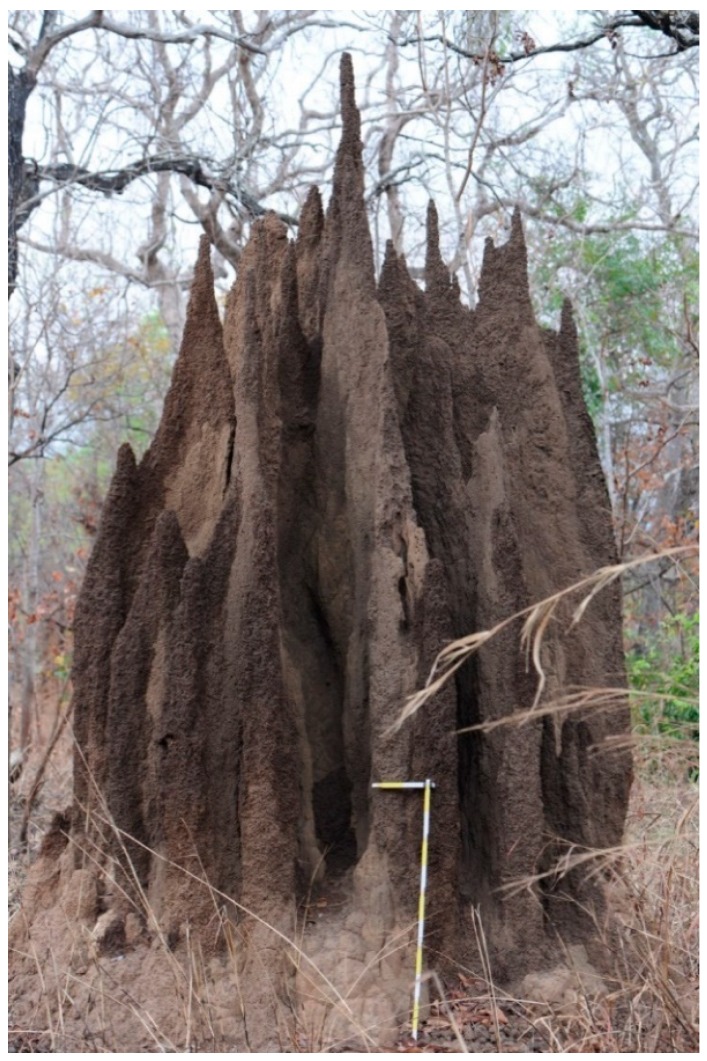
Termites do not only build charismatic mounds but also function as important ecosystem engineers in the tropics and subtropics.

Termites are major ecosystem engineers with crucial roles in decomposition, soil fertility, hydrology, and species diversity in tropical ecosystems (reviewed in: [1]). For instance, recent studies have shown that mound building species in African structure plants as well as animal communities, including those of other termites (e.g., [2,3]). In arid regions, the presence of termites and ants increases crop yield by 36% [4]. This knowledge has been traditionally applied in the Zaï system in semi-arid West Africa where termites are attracted to degraded land, which results in increased soil fertility, humidity and plant growth [5]. Successful application of this method leads to green savannah with shrubs and trees in an otherwise barren region. This is strikingly illustrated by the work of Yacouba Sawadogo, a farmer from Burkina Faso, who received the Right Livelihood Award in 2018 for his engagement in applying and improving the Zaï system. Concomitantly, however, termites have developed a bad reputation as pests by causing billions of dollars of damage annually [6,7]. However, only a few handfuls of species are pests, while 99% of the more than 2000 termite species offer beneficial ecosystem services [6,7]. Despite their importance, termites are largely understudied and most ecological research in tropical ecosystems concentrates on charismatic mound builders (Figure 1) while comparatively little is known about hypogeal termite species. Even species identities of important pest species of African crops are unclear (e.g., references [8,9]).

Studying termites is hampered by a lack of taxonomic knowledge because termites have few diagnostic morphological traits. Most identification is based on soldier characteristics but this generally allows the separation of genera only (e.g., reference [10]). Some species, especially soil feeding termites, can be identified by worker gut morphology [11,12]. Termites can have complex guts that serve as habitats for their symbiotic gut symbionts (mainly bacteria, but in lower termites also flagellates), which help, for instance, in digestion of plant material or nitrogen fixation (reviewed in: [13]). Gut morphology can be species specific, but not in all groups. For instance, fungus-growing termites (Macrotermitinae), which live in an obligate symbiosis with *Termitomyces* fungi, have very simplified guts that do not allow for species identification. However, they are the most common and dominant termites of African savannahs [14,15,16,17,18]. These taxonomical problems are large as revealed by a recent workshop on the taxonomy of West African termites (see below). Molecular identification can aid to overcome this problem, as sequencing of selected genes, mitochondrial genomes or entire genomes can help to reconstruct the phylogenetic relationships of species [19] and also delineate species (e.g., reference [20]). This has been also employed successfully in termites (e.g., references [14,21,22]), also including the most recently species delimitation approaches [23].

## 2. The Taxonomy of West African Termites as a Case

During a workshop held at the University of Freiburg (Germany) in April 2018, renowned researchers currently working with West African termites convened (author list, West African Termite Initiative). In order to test the reliability of species identification of West African termites, we applied the following procedure.

- Participants brought a sample collection of termites from their study areas which had been identified morphologically and/or using molecular markers. Thus, we had representative samples from Benin, Burkina Faso, Côte d’Ivoire, Guinea, Nigeria, Senegal, and Togo, covering the whole of West Africa.
- The samples were anonymized by giving them a simple letter/number code.
- Participants, including all world experts for West African termites, identified the samples using dissecting microscopes and their normal/standard keys and identification approach so that the same samples were repeatedly identified by different researchers.
- The identification results of the experts were compared to estimate the reliability of species identification.

The results of this approach were sobering. From about 70 species in total, only five were identified identically across experts: *Macrotermes bellicosus*, *Macrotermes subhyalinus*, *Fulleritermes tenebricus*, *Acanthotermes acanthothorax* and *Nasutitermes arborum* (Table S1). Researchers often identified even their own samples inconsistently. This illustrates the large ambiguity that exists in identification of West African termites, so that it may be warranted to speak about a West African taxonomy crisis.

Follow-up discussions of single species and combined knowledge by all experts finally (i) revealed some synonymous names (e.g., *Microtermes toumodiensis* = *Microtermes subhyalinus*), and (ii) resulted in the identification of species-diagnostic traits for several species, for instance the three *Ancistrotermes* species *Ancistrotermes cavithorax*, *Ancistrotermes crucifer* and *Ancistrotermes guineensis*, and three *Amitermes* species (*Amitermes evuncifer, A. guineensis* and *A. spinifer*) (Table S1). Consequently, we were able to unambiguously identify 18 species with their species-specific morphological characters and molecular barcoding sequence (in this case, COII) (Table S2) (category A ‘unambiguous species’ of the taxon status categories) (Table 1).

**Figure 2:**
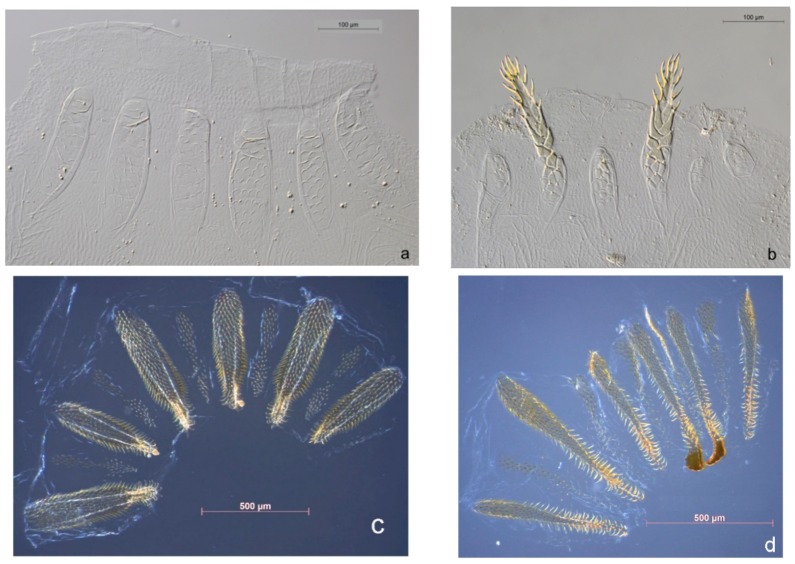
Example of contrasting enteric valve armature between closely related soil feeding termites. (**a**,**b**) Two species of soldier-less Apicotermitinae (*Astalotermes*-group) from Côte d’Ivoire. (**a**) *Astalotermes quietus*, (**b**) *Anenteotermes polyscolus*. Note the presence of six cushions in both species, all approximately equal and bearing crescent-shaped scales in *Ast. quietus*, but very dissimilar in *An. polyscolus* where two cushions bear long spiny extensions. Direction of flow is from bottom to top. (**c**,**d**): two Cubitermitinae from Côte d’Ivoire. (**c**) *Megagnathotermes notandus*: six primary cushions of almost equal lengths, bearing a mesial ridge and, on both sides, regularly-spaced long spines (kind of combs), narrow secondary cushions; (**d**) *Cubitermes proximatus*: two primary cushions bearing a sclerotized spatula sticking out of the valve, narrow secondary cushions.

For the remaining species, their states differ and we grouped them into two further taxon-status categories: category B ‘ambiguous species’ (i.e., species with some problems) and category C ‘critical taxa’ (i.e., taxa requiring major revisions’) (Table 1, Table S1). Category B includes all soil feeding termites. The best and very reliable approach to identify soil feeding species morphologically are gut dissections. Gut morphology reveals species-specific diagnostic markers such as the form and shape of the enteric valve (Figure 2). Revisions of soil feeding taxa are under way, such as for *Cubitermes* species (Josens & Deligne in prep). As expected, huge problems exist in the identification of the fungus-growing *Odontotermes* and *Microtermes* species, and e.g., species in the genus *Microcerotermes* (Table S1). Intensive work, including major taxonomical revisions, will thus be needed to fully resolve this (Category C). Surprisingly, however, *Trinervitermes* species (Nasutitermitinae) could also not be identified reliably even after intensive comparisons and discussions, and despite the existence of a widely-applied species-specific key [24]. Hence, this genus also requires revision and the species were also allocated to Category C (Table S1).

## 3. A Roadmap for Improving Termite Taxonomy

Based on the results of this workshop, we propose a set of guidelines for future termite taxonomy, also with the aim to extend and constantly update a world-wide online termite database, based on and in collaboration with the ‘Termite Catalog’ by R. Constantino (http://164.41.140.9/catal/) and the ‘Treatise on the Isoptera of the Word’ by K. Krishna, D. Grimaldi, V. Krishna, M.S. Engel and the American Museum of Natural History (http://digitallibrary.amnh.org). Besides species descriptions (see below), species distribution maps, identification keys, species-specific cuticular hydrocarbon profiles, and all literature available should be deposited in this database. Ideally, it should be supplemented with data for the fungal symbionts of fungus-growing termites (Macrotermitinae) and, where available, with information on associated gut symbionts. The database should be in English and, if possible, in French to facilitate use by West-African scientists.

### 3.1. Regional Taxonomic Status Workshops and A Global Termite Taxonomy Consortium

**Figure 3:**
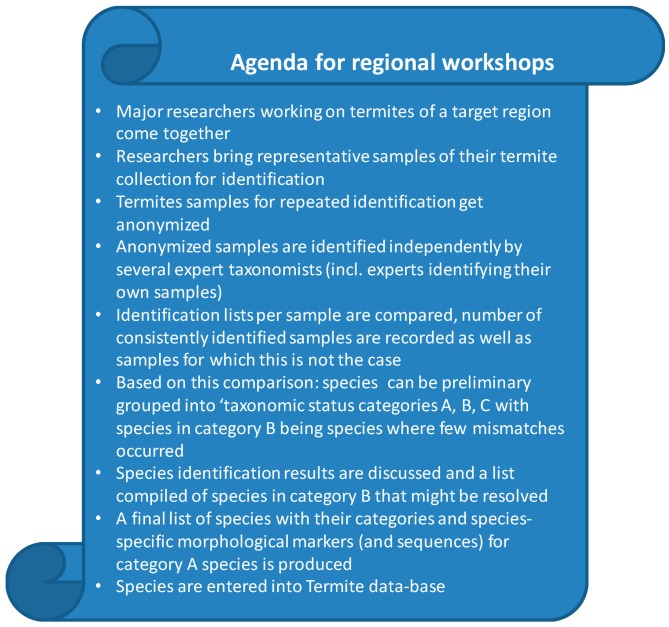
Proposal for a worldwide termite taxonomy initiative. Suggested procedure for regional taxonomy workshops.

In order to uncover how widespread taxonomical problems for different regions are, further region-specific workshops would be ideal, particularly if they apply comparable approaches to those for the West African case example. We suggest the procedure outlined in Figure 3. To further improve and standardize the procedure of sample re/identification (see above), a guideline can be developed which may specify, for instance, (i) the number of species to be re/identified; (ii) the regional coverage of the species; (iii) the number of experts who have to identify a specimen; and (iv) the follow-up discussion to narrow down species-specific diagnostic markers. These measures were not standardized during the West African termitologists’ workshop due to time constraints. Species should be grouped into A, B, C categories (Table 1).

In order to coordinate regional scale taxonomy workshops, workshops will be required with expert representatives from different geographical regions. The tasks of these “over-regional” workshops would be to first develop standardized workshop protocols and subsequently update, combine and synthesize regional efforts with the final aim to build up a reliable and standardized open-access termite database. This also requires the inclusion of bio-informaticians with data and/or computational analysis skills to organize and code the database. Such a multi-regional approach may also serve as a case example for other taxonomical groups. The taxonomic problems might be fewer in other regions without the diagnostic-poor Macrotermitinae subfamily. Despite the current increase in sequencing efforts, researchers without ready access to sequencing tools could use an open-access termite database that identifies and validates key morphological characters because most termite species still lack an accurate, useful morphological description.

### 3.2. Species Descriptions and Revisions 

Species descriptions should follow the general recommendations of the International Commission on Zoological Nomenclature (http://www.iczn.org). They should include a morphological description of the species together with the sequences of at least one marker gene/gene fragment. An iterative process between morphological and molecular analyses might be especially promising. Thus, the status of a species can be identified using morphological traits at the workshops. Then, sequencing efforts can help in species delimitation and guide further morphological analyses. For morphological descriptions, we recommend including as a minimum the following morphometrical measurements: e.g., head length and width; left hind-tibia length; left mandible length, on the ventral side; curvature of the mandibles; shape of gularmentum; shape of the labrum (see Table 2; for imagoes/alates see Table 3). These measurements need to be standardized. The morphometrical descriptions should be accompanied by photos and/or drawings and any genetic information should be included. As COII is the most reliable marker in terms of amplification success and taxonomic resolution [14,25,26], we recommend that at least this sequence should be published/deposited in NCBI but more sequences are recommended. Nuclear genes should be included to provide not only the information on the maternal species lineages. However, we recognize that they are often too conserved to provide good resolution at the species level. With the progress of next generation sequencing technologies, sequence availability will certainly become less of a limiting factor.

## 4. Perspectives Ahead

Termite taxonomy faces problems that hinder scientific progress. By implementing regional workshops integrated into a global termite taxonomy initiative (Figure 3), these problems can be overcome and lead to a continuously-updated open-access termite database. Such an initiative offers unpreceded opportunities, because (i) morphology is required for field-based identification; (ii) single-gene sequencing is becoming increasingly accessible for researchers, including in developing countries; and (iii) next-generation sequencing technologies will aid to resolve taxonomic problems and allow for combined termite and symbiont information. Additionally, the worldwide accessibility of the internet—also in developing countries—allows for maximal usage of the data. Thus, this initiative may also serve as a model for other taxa.

## 5. Conclusions

We identified major taxonomical problems for West African termites and provided a comprehensive termite species list for this region. Species are classified according to their taxonomic status category as category A ‘unambiguous species’, category B ‘ambiguous species’ (i.e., species with some problems) and category C ‘critical taxa’ (i.e., taxa requiring major revisions’). We suggest a roadmap for a worldwide termite taxonomy initiative based on regional workshops with standardized procedures to identify critical termite taxa and build up a common, open-access termite database. 

## Figures and Tables

**Table 1:** Taxonomic status categories (category A, B, C).

Category A ‘Unambiguous Species’	Category B ‘Ambiguous Species’	Category C ‘Critical Taxa’
Unambiguous species-specific morphological traits that have been defined & published	Species which have some difficulties but seem to have reliable morphological markers	Species/taxa with unclear status as, e.g., no reliable morphological marker exists, species descriptions are not published, synonymous names may exist
Molecular sequences for at least COII deposited in NCBI	They are currently revised and can relatively easily be revised	Revisions are difficult; they e.g., require studying a whole genus
	Molecular sequences generally lacking	Molecular sequences generally lacking

**Table 2:** List of minimum morphometric traits to measure on a soldier for species description.

Traits to Measure on Soldiers
number of antennal segments
head length and width
left hind-tibia length
left mandible length, on the ventral side
left mandible curvature
distance between first and second marginal teeth (on both mandibles)
distance between apical tooth and first marginal (on both mandibles)
gulamentum shape
labrum shape
enteric valve shape of workers (in case of a humivorous species)

**Table 3:** List of minimum morphometric traits to measure on an imago for species description.

Traits to Measure on Imagoes
head width
lesser and larger diameters of eyes and of ocelli
fontanelle shape
left hind-tibia length

## Supplementary Materials

The following are available online at http://www.mdpi.com/2075-4450/10/1/32/s1, Table S1: Taxonomic status of species occurring in West Africa. A. Species of major groups (sorted by family and subfamily) B. Miscellaneous single species from species poor taxa. The colour indicates the taxonomic status: green = category A: unambiguous species (species with defined species-specific morphological and genetic markers); yellow = category B: ambiguous species (species with problems but which are currently being addressed); red = category C: critical taxa (species that cannot be reliable identified), Table S2: Unambiguous species occurring in West Africa with species-specific morphological traits.
